# Ethnic differences in the patterns, incidence and prevalence of multiple long-term conditions: a systematic review protocol.

**DOI:** 10.12688/wellcomeopenres.25227.1

**Published:** 2026-04-10

**Authors:** Irene F. Bossman, Barbara I. Nicholl, Srinivasa Vittal Katikireddi, Susan Browne, Valerie Wells, Catherine A O'Donnell

**Affiliations:** 1 General Practice and Primary Care, University of Glasgow School of Health and Wellbeing, Glasgow, Scotland, UK; 2 Health Economics & Health Technology Assessment, University of Glasgow School of Health and Wellbeing, Glasgow, Scotland, UK; 3 University of Glasgow MRC/CSO Social and Public Health Sciences Unit, Glasgow, Scotland, UK

**Keywords:** Multiple long-term conditions (MLTC), multimorbidity, ethnicity, minority ethnic groups, ethnic inequalities, race, prevalence, multimorbidity patterns, multimorbidity types, systematic review

## Abstract

**Background:**

Multiple long-term conditions (MLTC) or multimorbidity is increasing among populations worldwide in the last decades. Less is known about the prevalence, socio-demographic patterning and combinations of long-term conditions among different ethnic groups globally. This systematic review protocol plans to review the current literature to understand the incidence, patterns, prevalence, and impact of multiple long-term conditions in different ethnic populations.

**Methods:**

This systematic review will include quantitative studies (cohort studies, cross-sectional, case-control, and baseline data from Randomised Control Trials) reporting on the patterns (combinations) and prevalence of multiple long-term conditions (MLTC) among adults across different ethnic groups in all settings (primary care, community or hospital). The following databases will be searched: Ovid MEDLINE, EMBASE, PsycINFO, CINAHL, ASSIA and COCHRANE CENTRAL using a combination of search terms including MLTC or multimorbidity and ethnicity. The exposure of interest is ethnicity, and included studies will report on at least one of these outcomes by ethnicity or a proxy of it: 1. prevalence and incidence of MLTC; 2. MLTC types, (count or patterns of conditions including physical-only, mental and physical and complex multimorbidity) and 3. health related outcomes (healthcare utilisation and mortality). Two reviewers will screen potential articles by titles/abstracts and full texts independently. A synthesis without meta-analysis approach to comprehensively summarise the key attributes and findings reported from each included study will be employed initially. Meta-analysis will be conducted where data allow, to estimate the risk of MLTC in minority ethnic groups vs majority ethnicity.

**Discussion:**

Some studies suggest people from ethnic minority backgrounds have a greater burden of MLTC, yet they are usually underserved in health research. Synthesising primary studies that focus on MLTC and ethnicity in this review will provide a global overview of prevalence or incidence data and their reported health outcomes among diverse ethnic populations.

## Plain Language Summary

Some people live with two or more long-term illnesses. We call this having multiple long-term conditions, or MLTC for short. Research shows that people with MLTC often experience worse overall health and well-being. The effects of MLTC can vary among different ethnic groups. For example, people from certain minority ethnic groups (like South Asian) are more likely to develop long-term conditions like Type 2 diabetes compared to the white majority ethnic group in the UK. However, we don’t know enough about how MLTC affect people from different ethnic groups.

To answer these questions, our study will be looking at all the existing research on this topic to understand which MLTC are common among people from different ethnic groups. We will find out the following:

1. Which combinations of illnesses are most frequent in minority ethnic people?
2. What factors might be related to these MLTC?
3. How often do people living with MLTC need hospital care?
4. Do MLTC affect how long minority ethnic people live?

## Introduction

### Background/rationale

In the past two decades, there has been increasing interest in multiple long-term conditions (MLTC), also known as, multimorbidity. ^1^ One in three adults globally is living with two or more long-term conditions, and in high-income countries like the United Kingdom (UK), the proportion of people living with four+ chronic conditions is likely to double in the next decade. ^2^ MLTC is increasingly prevalent with age but has an early onset among people from minority ethnic backgrounds and socioeconomically deprived areas. ^3, 4^ Measuring MLTC includes using disease counts to identify common clusters of conditions, or using validated weighted measures to aid in predicting patient outcomes and challenges associated with healthcare use. ^5^ Different types or patterns of MLTC exist, which is usually explained as the grouping of chronic diseases into different combinations based on the associations between them. ^6^ Most studies identified common patterns of MLTC to include cardiometabolic conditions, mental-health conditions, pulmonary and painful conditions in the general population. ^7, 8^

Though recent MLTC research focuses on understanding prevalence of MLTC and how they vary by age. sex and socio-economic position (SEP), there have been limited studies of MLTC focusing on ethnic variations. However, describing a population’s health in terms of ethnicity provide insights to disease causes affecting a particular ethnic group and help in designing targeted interventions and policies for health service planning. Commonly, ethnicity denotes a group of people with identities based on a mix of cultural and other shared factors such as ancestry, language, religion, diet, traditions and physical features traditionally associated with race. ^9^ The diversity in terms of socio-demographics, behavioural, cultural and other characteristics among ethnic groups accounts for differences in the health patterns of minority ethnic groups versus majority ethnic groups. ^10^ However, a complex interplay of factors such as deprivation, health-related behaviours, ‘healthy migrant effect’, racism and discrimination are also suggested causes for ethnic inequalities in health. The COVID-19 pandemic has revealed that people from minority ethnic groups experiences worst health outcomes, ^11^ placing an urgent call to health services including the UK National Health Service (NHS) to address these prevailing ethnic inequalities. ^12^

In conducting this review, we seek to identify the evidence base to deepen our understanding of the common combinations of long-term conditions that affect people from different ethnic groups modified by age, sex and SEP. A previous review by Busija and colleagues (2019) identified replicable and clinically meaningful patterns of MLTC but did not focus on classifying these patterns by ethnic groups. ^7^ Another relevant systematic review by Hayanga, Stafford and Becares (2023) has examined the prevalence of MLTC across ethnic groups in the UK only prior to the COVID-19 pandemic. ^13^ Their review provided a partial picture of ethnic inequalities in the prevalence of MLTC, suggesting that people who identify as South Asian (Indians, Pakistanis and Bangladeshi), Black (African, Caribbean and Other) and other Asian may be at increased risk of MLTC.

There is a complex pattern of MLTC accumulation and trajectories among people from minority ethnic backgrounds in studies conducted in the United States (US). An example is the study by Quinones *et al*. (2011) that reported health differences in the level and rate of multimorbidity changes among Black and Mexican Americans compared to White Americans during a 11-year study period. Black Americans aged 51 and older showed an elevated level of multimorbidity, though at a slower rate of increase compared to White Americans. ^14^

Together, these findings suggest that individuals from ethnic minority groups may have an early MLTC onset, different mixes of mental and physical chronic conditions or complex multimorbidity (three or more chronic conditions across three or more body systems) and possibly experience poorer health outcomes at least in high-income countries. Thus, there is an urgent need to better understand ethnic differences in the patterns and impact of MLTC. This paper outlines the protocol for a systematic review which aims to understand the patterns, prevalence, and incidence of MLTC, as well as associated health outcomes including healthcare utilisation and mortality across ethnic populations across the globe.

### Research questions

The aim of this review will be to systematically explore observational studies that present data on patterns of multiple long-term conditions (MLTC) among adults of different ethnic populations. The key research questions are:

1. How do the incidence and prevalence of MLTC vary across ethnic populations?
2. Is the association between ethnicity and MLTC incidence or prevalence modified by:
  a. age
  b. sex
  c. socio-economic position (SEP)
3. What are the common types of MLTC reported across different ethnic populations?
4. Does the risk of future healthcare utilisation and mortality following MLTC differ by ethnicity?

## Protocol methods

This systematic review protocol is registered with the international database of prospectively registered systematic reviews (PROSPERO CRD420251000101) and reported according to the Preferred reporting items for systematic review and meta-analysis protocols (PRISMA-P). ^15^

### Patient and Public Involvement and Engagement (PPIE)

PPIE advisors in our multimorbidity PhD programme with research expertise, together with PPIE members from diverse ethnic backgrounds have provided input that has shaped the research objectives from the start of this doctoral study. The PPIE members reviewed this systematic review objectives and reviewed the Plain Language Summary.

The current PPIE members with lived experience of MLTC or as caregivers for people with MLTC were recruited from community groups like the African and Caribbean Women’s Association in Scotland, and the Scottish Asylum Seekers Residents Association and some charity organisations for migrants in Glasgow. Discussions will continue as appropriate either in-person or virtually with this PPIE group at least thrice a year to contribute to the various aspects of this MLTC and ethnicity-focused study packages.

### Eligibility criteria

The eligibility criteria are formulated based on the population, exposure, comparator, outcome and study design (PECOS) format, an adapted version of the PICOS framework from the Cochrane Handbook, where ‘I’ (intervention) is substituted with ‘E’ (exposure). ^16^ The PECOS format is recommended in systematic reviews of observational studies, ^17, 18^ and it is employed in this review’s inclusion and exclusion criteria summarised in Table 1.

**Table 1.** PECOS inclusion and exclusion criteria.

PECOS	Inclusion criteria	Exclusion criteria
**Population**	A study where majority of the study population are adults (aged 18 and above) living with multiple long-term conditions (MLTC). MLTC is defined as the co-occurrence of two or more long-term conditions (either a physical non-communicable disease, a mental health condition, or an infectious disease of long duration) within an individual. Setting: Primary care, community or hospital settings will be considered	Studies where majority of the population are below 18 years Studies that recruited sample based on the presence of any specific condition or index diseases (e.g., diabetes) or complication of the single condition (that is, studies of comorbidity)
**Exposure**	Ethnicity: This will include studies with closely related indicators, (i.e. race, country of birth, migrant status, indigenous status or aboriginal groups).	Studies not reporting findings disaggregated by ethnicity will be excluded
**Comparator**	A comparator group is not relevant to this review, however, studies comparing different ethnic groups (at least two ethnicities) will be included.	Studies reporting on one ethnicity will be excluded
**Outcomes of interest or condition**	A study reporting on at least one of these outcomes by ethnicity or proxy below. Primary outcomes: 1. prevalence and incidence of MLTC, 2. MLTC types, (complex MLTC, mental and physical MLTC 3. health related outcomes (healthcare utilisation and mortality)	Studies that do not report on any of the outcomes. Comorbidity outcomes, where an outcome of a specified condition or set of conditions was reported relative to an index condition.
**Study Design**	Quantitative studies (cohort studies, cross-sectional, case-control, and baseline data from Randomised Control Trials) reporting on the patterns (combinations) and number of long-term conditions. Studies written in English	Animal studies Qualitative data, ecological studies, experimental studies Review studies will not be included. Conference papers, commentaries or editorials, opinion articles Pre-prints, retracted papers Studies not written in English

***Population***

This review will focus on adults living with MLTC in all settings (primary care, community or hospital). MLTC has been defined as the coexistence of two or more chronic health conditions in the same individual without any emphasis on a single condition as the primary focus; It is different from ‘comorbidity’, which describes the combined effects of added conditions in relation to an index chronic condition. ^19, 20^ An example is a study focusing on comorbidities of diabetes mellitus such as depression and stroke. This distinction between comorbidity and MLTC also known as multimorbidity led to the creation of a MeSH for ‘multimorbidity’ in January 2018. ^21^ Therefore, we will exclude studies that recruited their sample based on the presence of any specific condition or index disease (e.g., diabetes) or complication of the single condition (that is, studies of comorbidity). LTCs

This review will include studies where majority of the study population are adults (aged 18 and above) living with two or more chronic conditions and have data on ethnic groups. For studies assessing incidence and prevalence, some of the people may have MLTC while others may not have. However, prognostic studies will include all people living with MLTC.

***Exposure***

The exposure of interest is ‘ethnicity’. There is no international consensus on harmonised ethnicity classification to use in health research. However, due to the complexity of ethnicity and with some countries not collecting ethnicity data (including legal restrictions on collection), ^22^ studies with closely related indicators such as race, country of birth, migrant status, indigenous status or aboriginal groups will be included to help identify as much literature as possible. ^23^ Studies not reporting findings disaggregated by ethnicity will be excluded.

***Comparator***

Studies to be included need to have data available for comparison across at least two (or more) different ethnic groups.

***Outcome***

For this review, MLTC or multimorbidity is defined in accordance with the Academy of Medical Sciences as the co-occurrence of two or more long-term conditions (LTCs), (either a physical non-communicable disease, a mental health condition, or an infectious disease of long duration) within an individual. ^24^ Studies will be eligible if multimorbidity or MLTC are a central focus, regardless of data source used to assess MLTC status (e.g. self-report, healthcare records, or clinical assessments). Included studies may report on any of the following outcomes defined by ethnicity or proxy: prevalence or incidence of MLTC, MLTC types or patterns, and health related outcomes (healthcare utilisation and mortality) associated with MLTC.

Studies will be excluded if they report any of the outcomes including the incidence or prevalence of MLTC in relation to a single predefined index condition, without examining MLTCs in general.

In addition to the stated outcomes, we may extract and report other multimorbidity-related outcomes captured within the included studies such as health -related quality of life, mental health outcomes, activities of daily living and physical function. ^25^

**Definitions of MLTC types**

MLTC types refer to the grouping of chronic diseases or long-term conditions (LTCs) into different combinations or patterns based on the associations between them. ^6^ Therefore, MLTC types in this study will mean LTCs counts and patterns. We aim to identify MLTC types that report physical only MLTC, the mix of mental and physical LTCs and complex multimorbidity. Physical-only multimorbidity will be defined as two or more long-term physical conditions (e.g., diabetes + chronic obstructive pulmonary disease) coexisting within an individual. ^26^ Mental-physical multimorbidity will be defined as the co-occurrence of at least one mental health LTC (e.g. anxiety) and at least one physical health LTC (e.g. asthma) within the same individual. ^27^

Some authors have proposed the concept of complex multimorbidity to mean the “co-occurrence of three or more chronic conditions affecting three or more different body systems within one person, without defining an index condition”. ^28^ For this review, Harrison and colleagues’ definition of complex multimorbidity will be used. Other researchers suggest that a mixture of mental and physical conditions imply complexity, and that they are associated with greater adverse effect on health-related outcomes. ^29, 30^

***Study designs***

This review will include quantitative studies (cohort, cross-sectional, case-control, and baseline data from randomised control trials) reporting on the patterns (combinations) and number of long-term conditions. We will exclude the following: qualitative data, conference papers, commentaries or editorials and opinion articles. Only full text published articles will be included and studies not written in English will also be excluded.

### Study identification

***Data sources***

The following databases will be search using a combination of keywords and subject headings; Ovid MEDLINE, EMBASE, PsycINFO, CINAHL, ASSIA and COCHRANE CENTRAL. Authors will be contacted for further information where appropriate. Grey literature will not be searched in this review.

***Search strategy***

An initial scoping search was done using search terms ‘ethnicity’ and ‘multimorbidity’ adapted from two recent global systematic reviews focusing on: ethnic inequalities in COVID-19 infection and associated outcomes. ^11^; and a global and regional prevalence of multimorbidity in the adult population. ^31^ Boolean operators “AND” and “OR” will be used for additive and restrictive combinations of search terms where necessary. The search strategy development is supported by our college’s certified information specialist. Databases will be searched from their inception (e.g. Ovid MEDLINE ALL1946, EMBASE1947) to 9 ^th^ November 2025. A sample search strategy planned for use in Ovid-MEDLINE and for adaptation to all other databases is presented in Table 2.

**Table 2.** Ovid MEDLINE (R) example preliminary search strategy.

#	Searches
1.	Multiple Chronic Conditions/
2.	Multimorbidity/
3.	Multimorbidity.ab,kf,ti.
4.	"Multipatholog*”. ab,kf,ti.
5.	(multiple adj2 (problem* or condition* or illness* or chronic or mental or physical or long-term or long term)).ab,kf,ti.
6.	"Pluripatholog*”. ab,kf,ti.
7.	"Polymorbid*”. ab,kf,ti.
8.	1 or 2 or 3 or 4 or 5 or 6 or 7
9.	Minority Groups/
10.	Minority Health/
11.	”Emigrants and Immigrants”/
12.	”Ethnic and Racial Minorities”/
13.	Racial Groups
14.	Black People/ or Asian People/ or Roma /
15.	(ethnic* or asylum seeker* or BME or BAME or black or migrant* or minority or multiracial or refugee* or race or racial or cultur* or divers* or other white). ab,kf,ti.
16.	(ancestry adj2 (european or american or oceanic or asian or africa*)). ab,kf,ti.
17.	(Arab* or Africa* or Afro* or Asian or Bangladesh* or Caribbean or China or Chinese or India* or Irish or Pakistan*). ab,kf,ti.
18.	(americans adj2 (asian or hispanic or africans)). ab,kf,ti.
19.	indigenous.ab,kf,ti. or Indigenous Peoples/
20.	9 or 10 or 11 or 12 or 13 or 14 or 15 or 16 or 17 or 18 or 19
21.	8 AND 20

***Study selection***

Following the search, all identified studies will be exported to Endnote, a reference management software for deduplication, after which they will be uploaded into ‘DistillerSR’ a software management system for literature collection and screening purposes. A further check for duplicates will be conducted. All articles will be selected based on the inclusion and exclusion criteria. Two reviewers will screen potential articles by titles and abstracts independently to identify studies of potential relevance, with a low threshold for taking forward to the next stage. Again, the full text of selected studies will be assessed against the inclusion criteria by two independent reviewers, and any reasons for exclusion at this stage will be recorded.

Where conflicts arise between reviewers, this will be resolved through discussion, or with a third reviewer.

***Data extraction***

All studies after full text that meet inclusion criteria will pass to the data extraction stage. Data extraction will be done using Microsoft Excel and the following data extracted: general and study characteristics (e.g. author, title, publication year, country, study aim, study design, sample size, setting and data source); population characteristics (e.g., age, sex, ethnicity, socio-economic position); results and outcomes (e.g. definitions of MLTC, patterns of long-term conditions, type and number of chronic conditions, type of analysis, effect size, confounding factors (age, sex), health related outcomes, MLTC prevalence and incidence data) and other information (e.g. the conclusions of the author, links to relevant studies and information required for critical appraisal) will be extracted. Approaches on how included studies dealt with missing data will be reported and any sensitivity analyses performed to determine robustness of findings will be noted.

***Risk of bias (quality) assessment***

At least two reviewers will independently assess the risk of bias for each of the included studies, with any discrepancies resolved through discussion and, where required, the inclusion of a third reviewer. We will thoroughly review the quality of included studies using research quality assessment tools suited for observational studies, in this case, the Newcastle-Ottawa Scale (NOS) will be used. The NOS has been described as a simple, appropriate tool for quality assessment of non-randomised (observational) studies such as cohort and case-control studies. ^32^ The NOS will be further adapted to assess cross-sectional studies as used in some studies. ^33^ Study quality will not be used as an inclusion or exclusion criterion but would be used to ascertain the strength of the evidence, and whether it can threaten the conclusion of the exposure having a significant effect on the outcome.

***Data synthesis and analysis***

A synthesis without meta-analysis (SWiM) and quantitative synthesis (meta-analysis) methods will be employed to analyse the data. ^34^ Limitations to our synthesis plan will be made clear, and reasons will be provided for any revisions to the protocol on completion.

**General approach**

The study characteristics (author, year, country, study design), population details (age, sex, SEP and ethnicity), effect size, MLTC definitions, and the primary outcomes (incidence/prevalence, healthcare utilisation and mortality) will be extracted from each study, recorded, and presented in summary tables and figures. The specific synthesis plan in line with the research questions are documented below:

**Incidence and prevalence of MLTC across ethnic populations**

The incidence and prevalence rates data from included studies will be extracted. A forest plot will then be created comparing minority ethnic groups to the majority ethnic group, with estimates stratified by incidence vs prevalence and grouped by continent / country and ethnic group, with results ordered by year of study conduct.

Random-effects meta-analysis will be used to summarise prevalence estimates and estimate the risk of MLTC in minority vs majority ethnic group, as well as for each ethnic group compared to majority ethnicity in different countries.

Where estimates cannot be visually displayed on a forest plot, an effect direction plot will be created to display if risks were greater, similar or less in minority ethnic groups than the majority ethnicity.

**Effect modification by age, sex and SEP**

To determine whether the incidence or prevalence of MLTC differs by sex, age, and/or ethnicity, we will conduct subgroup analyses by stratifying sex estimates (men and women), age group estimates and SEP estimates. We will consider country/regional differences in the analyses. A random-effects meta-regression will investigate differences in associations with ethnicity by sex, age, socio-economic position and between studies on their outcome and quality assessment scores. Where meta-analysis is not feasible, findings will be compared across different settings using SWiM.

**Common types of MLTC across ethnic populations**

Reported types of MLTC will be summarised in a table reporting which combination of conditions have been found to be common in different ethnic groups, with results accounting for age and sex prioritised. Also, we will group findings by country/region and order findings by year where appropriate to capture country-specific differences. A narrative discussion will be provided to explain the differences.

**Ethnic differences in healthcare utilisation and mortality following MLTC development**

Pooled risk ratios (RR) and Hazard ratios (HR) or information on obtaining them will be calculated from included studies to assess risk of healthcare utilisation and mortality from having MLTC. Subgroups analyses by age, sex, SEP and different MLTC types (complex multimorbidity) Where possible, comparisons of effects for similar outcomes will be made across different ethnic groups.

***Heterogeneity assessment***

The variations between study results beyond those attributable to chance is known as statistical heterogeneity. ^35^ Heterogeneity among studies will be measured using the I ^2^ statistic to estimate the percentage of variation among the studies included in the review. Informal methods will be used to investigate heterogeneity in the findings when formal statistical investigation (subgroup analysis and meta-regression) is not possible. These methods could include using tables or tables to describe the synthesis methods.

***Meta-biases assessment***

Funnel plots will be used to evaluate publication bias. The Egger test may be used to test for funnel plot asymmetry. ^36^ Where there is bias, the graphical representation of funnel plots will often be skewed and asymmetrical. For the SWiM analysis, tables will be used to describe the synthesis of methods, limitations and subgroups of the review. ^34^

### Certainty of evidence

The overall strength of the body of evidence will be assessed using the Grading of Recommendations Assessment, Development, and Evaluation (GRADE) framework, considering factors such as study design, risk of bias, imprecision, inconsistency, indirectness and publication bias. ^37^ For GRADE, the certainty of evidence will be rated as high, moderate, low and very-low certainty. Using this systematic approach will ensure a comprehensive evaluation of the evidence base.

## Discussion

Ethnicity, as a multidimensional construct, is complex and influenced by genetic, environmental, socio-demographic, and healthcare-related factors. Previous research on single long-term conditions such as diabetes has reported ethnic inequalities across certain ethnic groups. As part of the recommended multimorbidity research priorities by the Academy of Medical Science report in 2018, ^24^ this systematic review will allow us to synthesise primary studies that focuses on MLTC and ethnicity, as well as, provide a global overview of prevalence or incidence data, and their reported health outcomes across ethnic groups. In addition, this review will provide insights into whether certain ethnic groups experience a higher burden of MLTC, and how these are influenced by age, sex, and socio-economic position.

The findings from this study can be essential for future resource planning and requirements for the provision of healthcare to people with MLTC.

### Strengths and limitations

A notable strength of this review is the inclusion of studies published globally. This will provide country-specific ethnic differences to the findings. However, this study may be limited by the expected heterogeneity across studies in terms of how MLTC are defined, measured, and reported. The differences in classifying ethnicity across different countries, healthcare access, and study designs may also impact the findings. To address these limitations, this study will employ a combination of meta-analysis (where feasible) and SWiM to ensure a robust and comprehensive review of the available evidence. Also, subgroup analyses and meta-regression will be conducted to explore potential effect modification by age, sex, and SEP.

## Conclusion

An urgent need to better understand the patterns of MLTC across ethnic groups aligns with global efforts to reduce health inequalities among different populations. This review may highlight the need for targeted interventions to address the unique challenges faced by minority ethnic groups, who are also reported to be underrepresented in intervention studies addressing MLTC. ^38^ There is potential to inform policy recommendations, and intervention strategies aimed at reducing ethnic health inequalities and improving health outcomes among different ethnic populations. This study will address gaps in the current literature, and guide future research in this critical area.

## Ethics and dissemination

This is a systematic review, so ethical approval is not needed. Results will be disseminated via peer-reviewed publication, conference presentations, patient and public involvement groups, professional networks and social media for engagement with the wider community.

## Data Availability

No underlying data is associated with this article. Figshare repository: Ethnic differences in the patterns, incidence and prevalence of multiple long-term conditions: a systematic review protocol. 10.6084/m9.figshare.31036987 Reference: [Bossman, Irene (2026). Ethnic differences in the patterns, incidence and prevalence of multiple long-term conditions: a systematic review protocol. figshare. Online resource. https://doi.org/10.6084/m9.figshare.31036987]. ^40^ This project contains the following extended data:
PRISMA-P Checklist is the Preferred reporting items for systematic review and meta-analysis protocols (PRISMA-P).
Table 1 is a summary of the PECOS (Population, Exposure, Comparator, Outcome and Study design) framework employed in the review’s inclusion and exclusion criteria.
Table 2 is a sample search strategy describing the search terms and keywords planned for use in Ovid-MEDLINE and for adaptation to all other databases. Data are available under the terms of the Creative Commons Attribution 4.0 International license (CC-BY 4.0). The study protocol is reported using the Preferred reporting items for systematic review and meta-analysis protocols checklist (PRISMA-P 2015). ^15^ Working DOI: https://doi.org/10.6084/m9.figshare.31036987 PRISMA-P checklist The search and study selection process of the main review will be conducted in accordance with the guidance provided in the Preferred Reporting Items for Systematic Reviews and Meta-analyses (PRISMA) statement. ^39^
