# Ethnic differences in the patterns, incidence and prevalence of multiple long-term conditions: a systematic review protocol.

**DOI:** 10.12688/wellcomeopenres.25227.3

**Published:** 2026-11-09

**Authors:** Irene F. Bossman, Barbara I. Nicholl, Srinivasa Vittal Katikireddi, Susan Browne, Valerie Wells, Catherine A O'Donnell

**Affiliations:** 1 General Practice and Primary Care, University of Glasgow School of Health and Wellbeing, Glasgow, Scotland, UK; 2 Health Economics & Health Technology Assessment, University of Glasgow School of Health and Wellbeing, Glasgow, Scotland, UK; 3 University of Glasgow MRC/CSO Social and Public Health Sciences Unit, Glasgow, Scotland, UK

**Keywords:** Multiple long-term conditions (MLTC), multimorbidity, ethnicity, minority ethnic groups, ethnic inequalities, race, prevalence, multimorbidity patterns, multimorbidity types, systematic review

## Abstract

**Background:**

Multiple long-term conditions (MLTC), also known as multimorbidity and commonly defined as two or more long term conditions in the same individual, are increasingly prevalent worldwide.. Less is known about the prevalence, incidence, socio-demographic patterning and combinations of long-term conditions acrossethnic groups. This systematic review protocol aims to examine ethnic differences in MLTC patterns, prevalence, incidence and health outcomes.

**Methods:**

This systematic review will include quantitative studies (cohort studies, cross-sectional, case-control, and baseline data from Randomised Control Trials) reporting on MLTC among adults across different ethnic groups in all settings (primary care, community or hospital). The following databases will be searched: Ovid MEDLINE, EMBASE, PsycINFO, CINAHL, ASSIA and COCHRANE CENTRAL using a combination of search terms including MLTC or multimorbidity and ethnicity. The exposure of interest is ethnicity, and included studies will report on at least one of these outcomes by ethnicity or a proxy of it: 1. prevalence and incidence of MLTC; 2. MLTC types (combinations or patterns of conditions including physical-only, mental and physical and complex multimorbidity, which is three or more (3+) chronic conditions across 3+ body system), and 3. health related outcomes (healthcare utilisation and mortality). Two reviewers will screen potential articles by titles/abstracts and full texts independently. A synthesis without meta-analysis approach to comprehensively summarise the key attributes and findings reported from each included study will be employed initially. Meta-analysis will be conducted where data allow, to estimate the risk of MLTC in minority ethnic groups vs majority ethnicity.

**Discussion:**

Some studies suggest some people from ethnic minority groups may have a greater burden of MLTC, yet these groups are usually underserved in health research. Synthesising primary studies on MLTC and ethnicity will provide a global overview of ethnic differences in prevalence, incidence, patterns and associated outcomes.

## Plain Language Summary

**Why are we doing this research?:**

Some people live with two or more long-term conditions, such as diabetes, asthma or depression. This is called having multiple long-term conditions, or MLTC for short. Research shows that people with MLTC can have poorer overall health and wellbeing. However, it is not clear whether MLTC affects people from different ethnic groups in the same way.

We know that some long-term health conditions are more common in some ethnic groups than others. For example, research on individual long-term conditions has found that type 2 diabetes is more common among people from some ethnic groups, including people from South Asian backgrounds, than among people from the White ethnic group in the UK.

But we know much less about how many But we know much less about how many people from different people from different ethnic groups have two or more long-term health conditions at the same time. We also do not know enough about which conditions commonly occur together or whether people from different ethnic groups have different health outcomes.

Understanding these differences could help us identify inequalities in health and healthcare. It could also help researchers and healthcare professionals understand whether people from different ethnic groups with MLTC may need different types of support or care.

**What will we do?:**

We will review existing research to find out:

1. How common is it for people from different ethnic groups to have two or more health conditions?
2. Does the link between ethnicity and MLTC vary according to age, sex or people’s social and economic circumstances?
3. Which combinations of long-term health conditions are most common in different ethnic groups?
4. Do people from different ethnic groups with MLTC have different risks of needing healthcare or dying?

## Introduction

### Background/rationale

There has been increasing interest in multiple long-term conditions (MLTC), also known as, multimorbidity. ^1^ A recent global systematic review and meta-analysis estimated that more than one in three adults living in the community have two or more long-term conditions (pooled prevalence: 37.2%), although estimates vary across populations and study methods. ^2^ In England, the proportion of adults aged 65 years and older living with four or more chronic conditions was projected to almost double between 2015 and 2035. ^3^ Some evidence suggests that MLTC is increasingly prevalent with age, while its onset may occur earlier among people living in socioeconomically deprived areas and some minority ethnic groups. ^4^ ^,^ ^5^ Measuring MLTC can involve using disease counts to quantify disease burden, identifying common clusters or patterns of conditions, ^6^ or using validated weighted measures to aid in predicting patient outcomes and challenges associated with healthcare use. ^7^ Different types or patterns of MLTC exist, which are usually explained as the grouping of chronic diseases into different combinations based on the associations between them. ^8^ Previous studies have identified common patterns of MLTC including cardiometabolic conditions, mental-health conditions, pulmonary and painful conditions in the general population. ^6^ ^,^ ^9^

Though recent MLTC research focuses on understanding prevalence of MLTC and how it varies by age, sex and socio-economic position (SEP), there have been limited studies of MLTC focusing on ethnic variations. Commonly, ethnicity refers to shared identities based on a mix of cultural and other shared factors such as ancestry, language, religion, diet, traditions and physical features traditionally associated with race. ^10^ Examining population health by ethnicity can provide insights into the factors contributing to disease patterns within specific ethnic groups and can help inform targeted interventions and health service planning. The diversity in terms of sociodemographic, behavioural, cultural and other characteristics among ethnic groups accounts for differences in the health patterns of minority ethnic groups versus majority ethnic groups. ^11^ However, a complex interplay of factors such as deprivation, health-related behaviours, ‘the healthy migrant effect’, racism and discrimination may also contribute to ethnic inequalities in health. The COVID-19 pandemic has revealed that people from some minority ethnic groups experiences worse COVID-19 outcomes compared with white majority populations, ^12^ highlighting an urgent need to health services, including the UK National Health Service (NHS), to address these prevailing ethnic inequalities. ^13^

In conducting this review, we seek to identify the evidence base to deepen our understanding of the common combinations of long-term conditions that affect people from different ethnic groups and examine whether associations between ethnicity and MLTC prevalence or incidence vary by age, sex and SEP. A previous review by Busija and colleagues (2019) identified replicable patterns of MLTC; however, their synthesis did not focus specifically on classifying these patterns by ethnic group. ^6^ Another relevant systematic review by Hayanga, Stafford and Becares (2023) examined the prevalence of MLTC across ethnic groups in the UK general population, focusing on evidence before and during the very early stages of the COVID-19 pandemic. ^14^ Their review provided a partial picture of ethnic inequalities in the prevalence of MLTC, suggesting that people from South Asian (Indian, Pakistani and Bangladeshi), Black (African, Caribbean and Other), other Asian and mixed ethnicity groups may be at higher risk of MLTC than the majority white population. ^14^ A further review by Hayanga and colleagues examined ethnic inequalities in healthcare use and care quality among people with MLTC living in the UK, and found evidence suggesting inequalities in emergency admissions and disease management. ^15^

**Table 1.** PECOS inclusion and exclusion criteria.

PECOS	Inclusion criteria	Exclusion criteria
**Population**	Studies involving aged 18 years and above, where data for the adult population can be extracted separately. For incidence or prevalence studies, the population may include individuals with and without multiple long-term conditions (MLTC). For prognostic studies, the study population will include adults with MLTC. Setting: All settings including primary care, community or hospital settings will be considered.	Studies where the population is exclusively below 18 years, or where data for adults aged 18+ cannot be extracted separately. Studies in which ethnicity specific data for the relevant MLTC outcome cannot be extracted. Studies that recruited sample based on the presence of any specific condition or index diseases (e.g., diabetes) or complication of the single condition (that is, studies of comorbidity).
**Exposure**	Ethnicity: This will include studies with closely related indicators, (i.e. race, country of birth, migrant status, indigenous status or aboriginal groups).	Studies not reporting findings disaggregated by ethnicity will be excluded.
**Comparator**	A comparator group is not relevant to this review, however, studies comparing different ethnic groups (at least two ethnicities) will be included.	Studies reporting on one ethnicity will be excluded.
**Outcomes of interest or condition**	A study reporting on at least one of these outcomes by ethnicity or proxy below. Primary outcomes: 1. prevalence and incidence of MLTC, 2. MLTC types, (complex MLTC, mental and physical MLTC 3. health related outcomes (healthcare utilisation and mortality)	Studies that do not report on any of the outcomes. Comorbidity outcomes, where an outcome of a specified condition or set of conditions was reported relative to an index condition.
**Study Design**	Quantitative studies (cohort studies, cross-sectional, case-control, and baseline data from Randomised Control Trials) reporting on the patterns (combinations) and number of long-term conditions. Peer-reviewed articles	Animal studies Qualitative data, ecological studies, experimental studies Review studies will not be included. Conference papers, commentaries or editorials, opinion articles Pre-prints, retracted papers

Whether the patterns reported in the UK are consistent across other national contexts remains uncertain. Studies conducted in the United States (US) also indicate differences in MLTC levels and trajectories between racial and ethnic groups. For example, Quinones *et al*. (2011) examined differences in multimorbidity levels and rates of accumulation among Black and Mexican Americans compared to White Americans, following adults aged 51 years and older for up to 11 years. Black Americans showed higher levels of multimorbidity, but a slower rate of accumulation than White Americans, while Mexican Americans had lower levels and slower accumulation in adjusted analyses. ^16^

Together, these findings suggest that ethnic inequalities in MLTC are heterogenous, although their consistency across national setting remain unclear. ^14^ ^–^ ^16^ Thus, there is a need to better understand ethnic differences in the patterns and impact of MLTC globally. Building on Hayanga et al.’s UK-focused review of MLTC prevalence, our review will extend the geographical scope and examine ethnic differences in condition patterns, incidence and associated outcomes, alongside prevalence. ^14^ ^,^ ^15^ This paper outlines the protocol for a systematic review examining these differences globally, including associated healthcare utilisation and mortality.

### Research questions

The aim of this review is to systematically explore observational studies that present data on the prevalence, incidence and patterns of MLTC among adults of different ethnic groups. The key research questions are:

1. How do the incidence and prevalence of MLTC vary by ethnicity within populations?
2. Is the association between ethnicity and MLTC incidence or prevalence modified by:
  a. age
  b. sex
  c. socio-economic position (SEP)
3. What are the common types of MLTC reported across different ethnic groups?
4. Does the risk of future healthcare utilisation and mortality following MLTC differ by ethnicity?

## Protocol methods

This systematic review protocol is registered with the international database of prospectively registered systematic reviews (PROSPERO CRD420251000101) and reported according to the Preferred reporting items for systematic review and meta-analysis protocols (PRISMA-P). ^17^

### Patient and Public Involvement and Engagement (PPIE)

Our university based PPIE advisors with research expertise, supported community engagement and helped identify community and organisation leaders to support recruitment. A total of 10 PPIE members from diverse ethnic and migrant groups with lived experience of MLTC or as caregivers for people with MLTC were recruited from community groups like the African and Caribbean Women’s Association in Scotland, and the Scottish Asylum Seekers Residents Association and migrant support organisations in Glasgow.

PPIE members provided feedback on the review objectives and confirmed their relevance. They highlighted the importance of considering mental health, particularly anxiety and depression, as an integral component of MLTC. In response, the review will identify different types of MLTC and studies that include mental health conditions within their definition of MLTC. These findings will also inform subsequent related projects. PPIE members also reviewed the Plain Language Summary and provided feedback to improve its clarity.

### Eligibility criteria

The eligibility criteria are formulated based on the population, exposure, comparator, outcome and study design (PECOS) format, an adapted version of the PICOS framework from the Cochrane Handbook, where ‘I’ (intervention) is substituted with ‘E’ (exposure). ^18^ The PECOS format is recommended in systematic reviews of observational studies, ^19^ ^,^ ^20^ and it is employed in this review’s inclusion and exclusion criteria summarised in Table 1.

***Population***

This review will focus on adults aged 18 years and above (18+) with data on ethnicity and MLTC in all settings (including primary care, community or hospital). MLTC has been defined as the coexistence of two or more chronic health conditions in the same individual without any emphasis on a single condition as the primary focus; It is different from ‘comorbidity’, which describes the combined effects of added conditions in relation to an index chronic condition. ^21^ ^,^ ^22^ An example is a study focusing on comorbidities of diabetes mellitus such as depression and stroke. This distinction between comorbidity and MLTC also known as multimorbidity led to the creation of a MeSH for ‘multimorbidity’ in January 2018. ^23^ We will exclude studies that recruited their sample based on the presence of any specific condition or index disease (e.g., diabetes) or complication of the single condition (that is, studies of comorbidity).

For studies assessing incidence and prevalence of MLTC, the study population may include adults with and without MLTC, provided that the study reports the relevant outcomes by ethnic group or proxy. For prognostic studies, we will include all adults living with MLTC.

***Exposure***

The primary exposure of interest is ethnicity, recognising that ethnicity is socially constructed, multidimensional concept shaped by social, cultural and historical contexts. There is no international consensus on harmonised ethnicity classification to use in health research, and ethnic classifications vary across countries. However, as some countries do not routinely collect ethnicity data, including due to legal restrictions, ^24^ studies using closely related constructs such as race, Indigenous status or Aboriginal status, country of birth and migration status, will also be included. ^25^ Country of birth and migrant status will be treated as migration-background indicators rather than as equivalent to ethnicity, and findings based on these indicators will be synthesised separately. Studies not reporting findings disaggregated by ethnicity or race or another eligible related construct will be excluded.

***Comparator***

Studies to be included need to have data available for comparison across at least two (or more) different ethnic groups.

***Outcome***

For this review, MLTC or multimorbidity is defined in accordance with the Academy of Medical Sciences as the co-occurrence of two or more long-term conditions (LTCs), (either a physical non-communicable disease, a mental health condition, or an infectious disease of long duration) within an individual. ^26^ Studies will be eligible if multimorbidity or MLTC are a central focus, regardless of data source used to assess MLTC status (e.g. self-report, healthcare records, or clinical assessments). Included studies may report on any of the following outcomes defined by ethnicity or proxy: MLTC prevalence or incidence, MLTC types or patterns, and health related outcomes (healthcare utilisation and mortality) associated with MLTC.

Studies will be excluded if they report any of the outcomes including the incidence or prevalence of MLTC in relation to a single predefined index condition, without examining MLTCs in general.

In addition to the stated outcomes, we may extract and report other multimorbidity-related outcomes captured within the included studies such as health -related quality of life, mental health outcomes, activities of daily living and physical function. ^27^

**Definitions of MLTC types**

MLTC types refer to the grouping of chronic diseases or LTCs into different combinations or patterns based on the associations between them. ^8^ Therefore, MLTC types in this study will mean LTCs counts and patterns. We aim to identify MLTC types that report physical only MLTC, the mix of mental and physical LTCs and complex multimorbidity. Physical-only multimorbidity will be defined as two or more long-term physical conditions (e.g., diabetes + chronic obstructive pulmonary disease) coexisting within an individual. ^28^ Mental-physical multimorbidity will be defined as the co-occurrence of at least one mental health LTC (e.g. anxiety) and at least one physical health LTC (e.g. asthma) within the same individual. ^29^

Some authors have proposed the concept of complex multimorbidity to mean the “co-occurrence of three or more (3+) chronic conditions affecting 3+ different body systems within one person, without defining an index condition”. ^30^ For this review, Harrison and colleagues’ definition of complex multimorbidity will be used. Other researchers suggest that a mixture of mental and physical conditions imply complexity, and that they are associated with greater adverse effect on health-related outcomes. ^31^ ^,^ ^32^

***Study designs***

This review will include quantitative studies (cohort, cross-sectional, case-control, and baseline data from randomised control trials) reporting on the patterns (combinations) and number of long-term conditions. We will exclude the following: qualitative data, conference papers, commentaries or editorials and opinion articles. Only full text peer-reviewed published articles will be included. No language restrictions will be applied in the search, and studies not published in English will be translated where required.

### Study identification

***Data sources***

The following databases will be search using a combination of keywords and subject headings; Ovid MEDLINE, EMBASE, PsycINFO, CINAHL, ASSIA and COCHRANE CENTRAL. These databases were selected to provide complementary coverage of biomedical, social science, psychological, nursing and allied health literature relevant MLTC and ethnicity research. Authors will be contacted for further information where appropriate. Grey literature will not be searched in this review.

***Search strategy***

An initial scoping search was done using search terms ‘ethnicity’ and ‘multimorbidity’ adapted from two recent global systematic reviews focusing on: ethnic inequalities in COVID-19 infection and associated outcomes ^12^; and a global and regional prevalence of multimorbidity in the adult population. ^2^ Boolean operators “AND” and “OR” will be used for additive and restrictive combinations of search terms where necessary. The search strategy development is supported by our college’s certified information specialist. Databases will be searched from their inception (e.g. Ovid MEDLINE ALL1946, EMBASE1947) to 9 ^th^ November 2025. A sample search strategy planned for use in Ovid-MEDLINE and for adaptation to all other databases is presented in Table 2.

**Table 2.** Ovid MEDLINE (R) example preliminary search strategy.

#	Searches
1.	Multiple Chronic Conditions/
2.	Multimorbidity/
3.	Multimorbidity.ab,kf,ti.
4.	"Multipatholog*”. ab,kf,ti.
5.	(multiple adj2 (problem* or condition* or illness* or chronic or mental or physical or long-term or long term)).ab,kf,ti.
6.	"Pluripatholog*”. ab,kf,ti.
7.	"Polymorbid*”. ab,kf,ti.
8.	1 or 2 or 3 or 4 or 5 or 6 or 7
9.	Minority Groups/
10.	Minority Health/
11.	”Emigrants and Immigrants”/
12.	”Ethnic and Racial Minorities”/
13.	Racial Groups
14.	Black People/ or Asian People/ or Roma /
15.	(ethnic* or asylum seeker* or BME or BAME or black or migrant* or minority or multiracial or refugee* or race or racial or cultur* or divers* or other white). ab,kf,ti.
16.	(ancestry adj2 (european or american or oceanic or asian or africa*)). ab,kf,ti.
17.	(Arab* or Africa* or Afro* or Asian or Bangladesh* or Caribbean or China or Chinese or India* or Irish or Pakistan*). ab,kf,ti.
18.	(americans adj2 (asian or hispanic or africans)). ab,kf,ti.
19.	indigenous.ab,kf,ti. or Indigenous Peoples/
20.	9 or 10 or 11 or 12 or 13 or 14 or 15 or 16 or 17 or 18 or 19
21.	8 AND 20

***Study selection***

Following the search, all identified studies will be exported to Endnote, a reference management software for deduplication, after which they will be uploaded into ‘DistillerSR’ a software management system for literature collection and screening purposes. A further check for duplicates will be conducted. All articles will be selected based on the inclusion and exclusion criteria. Two reviewers will screen potential articles by titles and abstracts independently to identify studies of potential relevance, with a low threshold for taking forward to the next stage. Again, the full text of selected studies will be assessed against the inclusion criteria by two independent reviewers, and any reasons for exclusion at this stage will be recorded.

Where conflicts arise between reviewers, this will be resolved through discussion, or with a third reviewer.

***Data extraction***

All studies after full text that meet inclusion criteria will pass to the data extraction stage. Data extraction will be done using Microsoft Excel and the following data extracted: general and study characteristics (e.g. author, title, publication year, country, study aim, study design, sample size, setting and data source); population characteristics (e.g., age, sex, ethnicity, socio-economic position); results and outcomes (e.g. definitions of MLTC, patterns of long-term conditions, type and number of chronic conditions, type of analysis, effect size, confounding factors (age, sex), health related outcomes, MLTC prevalence and incidence data) and other information (e.g. the conclusions of the author, links to relevant studies and information required for critical appraisal) will be extracted. Where reported and relevant, approaches on how included studies dealt with missing data will be reported and any sensitivity analyses undertaken will be noted.

***Risk of bias (quality) assessment***

At least two reviewers will independently assess the risk of bias for each of the included studies, with any discrepancies resolved through discussion and, where required, the inclusion of a third reviewer. We will thoroughly review the quality of included studies using research quality assessment tools suited for observational studies, in this case, the Newcastle-Ottawa Scale (NOS) will be used. The NOS has been described as a simple, appropriate tool for quality assessment of non-randomised (observational) studies such as cohort and case-control studies, particularly in studies evaluating associations rather than demonstrating causality. ^33^ The NOS will be further adapted to assess cross-sectional studies as used in some studies. ^34^ Risk of bias scores will not be used as an inclusion or exclusion criterion but will inform confidence in the findings and the interpretation of associations between the exposure and outcomes.

***Data synthesis and analysis***

A synthesis without meta-analysis (SWiM) and quantitative synthesis (meta-analysis) methods will be employed to analyse the data. ^35^ SWiM will be reported using relevant items from the SWiM guideline. Meta-analysis will be undertaken where at least two studies are considered sufficiently comparable in terms of ethnicity or related indicator construct, multimorbidity definition, and reported outcome measures. Limitations to our synthesis plan will be made clear, and reasons will be provided for any revisions to the protocol on completion.

**General approach**

The study characteristics (author, year, country, study design), population details (age, sex, SEP and ethnicity), effect size, MLTC definitions, and the primary outcomes (incidence/prevalence, healthcare utilisation and mortality) will be extracted from each study, recorded, and presented in summary tables and figures. We will describe how ethnicity and related constructs are conceptualised, and how MLTC is defined, measured and classified, including the conditions considered, diagnostic thresholds, and methods of ascertainment in the included studies. This information will be used to contextualise the synthesis of ethnic differences in MLTC prevalence, incidence and associated outcomes, and to assess the comparability of finding across studies. The specific synthesis plans, aligned with the review questions, are outlined below:

**Incidence and prevalence of MLTC across ethnic populations**

Incidence and prevalence estimates will be extracted from included studies. Where suitable comparative estimates and measures of uncertainty are available, forest plots will compare minority ethnic groups to the majority ethnic group or other specified reference group. Incidence and prevalence findings will be presented separately, grouped by continent or country and ethnic comparison, with results ordered by year of study conduct. Estimates adjusted for age and sex will be prioritised. Where multiple estimates are reported, selection will consider adjustment sets and follow-up duration, while avoiding double-counting participants within the same synthesis.

Random-effects meta-analysis will summarise prevalence estimates and, separately, associations between ethnicity and MLTC occurrence, comparing minority or individual ethnic groups with the majority ethnic group in different countries, where studies are sufficiently comparable.

Where estimates cannot be presented appropriately in a forest plot, effect direction plots will be considered to display whether MLTC occurrence is higher or lower in minority ethnic groups than in the reference group. Uncertainty in the estimates will be considered when interpreting the direction of associations.

**Effect modification by age, sex and SEP**

To determine whether associations between ethnicity and MLTC incidence or prevalence differs by sex, age, and/or SEP, we will conduct subgroup analyses using sex-specific estimates (men and women), age-group estimates and SEP-specific estimates. We will consider country/regional differences in the analyses. Where there are comparable studies, a random-effects meta-regression will investigate ethnicity-related associations according to study-level characteristics, including sex, age, SEP measures and outcome definitions. Where meta-analysis is not feasible, findings will be synthesised narratively, grouping studies by outcome, ethnic comparison, setting and relevant subgroup characteristics. Reporting will follow relevant items from the SWiM guideline where applicable.

**Common types of MLTC across ethnic populations**

Reported types of MLTC will be summarised in a table showing which combinations are common across ethnic groups, with results accounting for age and sex prioritised where available. Also, we will group findings by country/region and order findings by year where appropriate to capture country-specific patterns. A narrative discussion will be provided to explain the differences between countries, regions and healthcare settings.

**Ethnic differences in healthcare utilisation and mortality following MLTC development**

Risk ratios (RR) and Hazard ratios (HR) or data sufficient to calculate them will be extracted from included studies to assess risk of healthcare utilisation and mortality among people with MLTC. Other relevant measures, including odds ratios and healthcare utilisation rates, will also be extracted and synthesised separately, with pooling considered where studies and measures are comparable. Subgroups analyses by age, sex, SEP and MLTC type, including (complex multimorbidity), will be conducted where sufficient data are available. Where possible, comparisons of effects for similar outcomes will be made across different ethnic groups.

***Heterogeneity assessment***

Variation between study results beyond those attributable to chance is known as statistical heterogeneity. ^36^ Heterogeneity among studies will be measured using the I ^2^ statistic to estimate the percentage of variation in study estimates attributable to heterogeneity rather than chance. Informal methods will be used to investigate heterogeneity in the findings when formal statistical investigation (subgroup analysis and meta-regression) is not possible. These methods could include using tables or narrative comparisons to describe differences in study characteristics and findings.

***Meta-biases assessment***

Funnel plots will be used to evaluate publication bias. The Egger test may be used to test for funnel plot asymmetry. ^37^ Where there is bias, the graphical representation of funnel plots will often be skewed and asymmetrical. For the SWiM analysis, tables will be used to describe the synthesis of methods, limitations and subgroups of the review. ^35^

### Certainty of evidence

The overall strength of the body of evidence will be assessed using the Grading of Recommendations Assessment, Development, and Evaluation (GRADE) framework, considering factors such as study design, risk of bias, imprecision, inconsistency, indirectness and publication bias. ^38^ For GRADE, the certainty of evidence will be rated as high, moderate, low and very-low certainty. Using this systematic approach will ensure a comprehensive evaluation of the evidence base.

## Discussion

Ethnicity is a complex, multidimensional construct, and ethnic differences in health may reflect environmental, socio-demographic, and healthcare-related factors. In line with the multimorbidity research priorities recommended by the Academy of Medical Sciences’ 2018 report, ^26^ this systematic review will synthesise primary studies that focuses on MLTC and ethnicity, as well as provide a global overview of prevalence, incidence, and associated outcomes across ethnic groups. This review will provide further insights into whether certain ethnic groups experience a higher burden of MLTC and whether associations between ethnicity and MLTC prevalence or incident vary by age, sex, and SEP.

The findings from this study may inform future resource planning and healthcare provision for people with MLTC.

### Strengths and limitations

A notable strength of this review is the inclusion of studies published globally. This will provide insights into country-specific ethnic differences where evidence is available. However, this review may be limited by heterogeneity across studies in terms of how MLTC are defined, measured, and reported. Differences in classifying ethnicity across different countries, healthcare access, and study designs may also impact the findings. To account for these limitations, this review will employ a meta-analysis where feasible and structured synthesis without meta-analysis where pooling is inappropriate, with relevant SWiM reporting items used to support transparent reporting. Also, subgroup analyses will explore whether associations between ethnic and MLTC prevalence or incidence vary by age, sex, and SEP where sufficient data are available, and meta-regression will explore between study variation where the data permit.

## Conclusion

The need to better understand the patterns of MLTC across ethnic groups aligns with global efforts to reduce health inequalities among different populations. This review may highlight the need for targeted interventions to address the specific challenges faced by minority ethnic groups, who are also reported to be underserved in intervention studies addressing MLTC. ^39^ The findings may inform policy recommendations, and intervention strategies aimed at reducing ethnic health inequalities and improving health outcomes across ethnic groups and related classification group. This review will identify gaps in the current literature, and guide future research in this area.

## Ethics and dissemination

This is a systematic review, so ethical approval is not needed. Results will be disseminated via peer-reviewed publication, conference presentations, patient and public involvement groups, professional networks and social media for engagement with the wider community.

## Data Availability

No underlying data is associated with this article. Figshare repository: Ethnic differences in the patterns, incidence and prevalence of multiple long-term conditions: a systematic review protocol. 10.6084/m9.figshare.31036987 Reference: [Bossman, Irene (2026). Ethnic differences in the patterns, incidence and prevalence of multiple long-term conditions: a systematic review protocol. figshare. Online resource. https://doi.org/10.6084/m9.figshare.31036987]. ^40^ This project contains the following extended data:
• PRISMA-P Checklist is the Preferred reporting items for systematic review and meta-analysis protocols (PRISMA-P). •
Table 1 is a summary of the PECOS (Population, Exposure, Comparator, Outcome and Study design) framework employed in the review’s inclusion and exclusion criteria. • Table 2 is a sample search strategy describing the search terms and keywords planned for use in Ovid-MEDLINE and for adaptation to all other databases. Data are available under the terms of the Creative Commons Attribution 4.0 International license (CC-BY 4.0). The study protocol is reported using the Preferred reporting items for systematic review and meta-analysis protocols checklist (PRISMA-P 2015). ^17^ Working DOI: https://doi.org/10.6084/m9.figshare.31036987 PRISMA-P checklist The search and study selection process of the main review will be conducted in accordance with the guidance provided in the Preferred Reporting Items for Systematic Reviews and Meta-analyses (PRISMA) statement. ^41^
