# Supplementary material for: Ethnic differences in the patterns, incidence and prevalence of multiple long-term conditions: a systematic review protocol
Source: Wellcome Open Res. 2026 Nov 9;11:215. Originally published 2026 Apr 10. [Version 3] doi: 10.12688/wellcomeopenres.25227.3 (PMC13598395; doi:10.12688/wellcomeopenres.25227.3)
Supplement: Peer review history

# Reviewer report: Reviewer response for version 3

**DOI:** 10.21956/wellcomeopenres.31135.r179133

**Published:** 2026-11-12

**Authors:** Rachel Cooper

**Affiliations:** 1 Newcastle University, Newcastle upon Tyne, England, UK

I thank the authors for carefully considering my previous comments and for addressing all of these satisfactorily in their revisions.

Is the study design appropriate for the research question?

Partly

Is the rationale for, and objectives of, the study clearly described?

Partly

Are sufficient details of the methods provided to allow replication by others?

Partly

Are the datasets clearly presented in a useable and accessible format?

Not applicable

Reviewer Expertise:

Inequalities in multiple long-term conditions; life course epidemiology

I confirm that I have read this submission and believe that I have an appropriate level of expertise to confirm that it is of an acceptable scientific standard.

# Reviewer report: Reviewer response for version 2

**DOI:** 10.21956/wellcomeopenres.30289.r166848

**Published:** 2026-09-28

**Authors:** Hilda Hounkpatin

**Affiliations:** 1 University of Southampton, Southampton, UK

This is an important area of research, and the protocol is well-written, methodologically sound with overall good level of detail and clarity. I have minor suggestions:

 The rationale of the study is good but could be further strengthened. Why is it important to understand whether variations in MLTC by ethnic groups vary globally? How will this review add to the review by Hyangana et al?

 It would be helpful to include a sentence justifying/explaining your choice of search databases.

 Table 1. I’m not sure country of birth alone is necessarily an accurate indicator of ethnicity. Consider removing this or justify including this.

Is the study design appropriate for the research question?

Yes

Is the rationale for, and objectives of, the study clearly described?

Partly

Are sufficient details of the methods provided to allow replication by others?

Yes

Are the datasets clearly presented in a useable and accessible format?

Yes

Reviewer Expertise:

epidemiology of long-term conditions, health inequalities, mixed methods research

I confirm that I have read this submission and believe that I have an appropriate level of expertise to confirm that it is of an acceptable scientific standard.

# Response

**Published:** 2026-11-02

**Authors:** Irene Bossman

**Affiliations:** School of Health and Wellbeing, University of Glasgow College of Medical Veterinary and Life Sciences, Glasgow, Scotland, UK

**Response: **Thank you for these helpful comments. We have strengthened the rationale for examining ethnic differences in MLTC across countries and regions. Differences in MLTC prevalence and patterns may reflect variation in population characteristics, migration background, socioeconomic circumstances, healthcare systems and environmental exposures. Understanding whether inequalities are consistent across settings is important for assessing whether findings can be generalised to other populations and informing context-sensitive prevention and health-service planning. We have also clarified how our review builds on Hayanga et al.’s UK-focused review of ethnic inequalities in MLTC prevalence. Our review extends the geographical scope and examines ethnic differences in MLTC patterns, prevalence, incidence and associated outcomes, including healthcare utilisation and mortality. It will also assess whether associations between ethnicity and MLTC prevalence or incidence vary by age, sex and socioeconomic position. The revised introduction makes these contributions clearer. It would be helpful to include a sentence justifying/explaining your choice of search databases.

**Response**: Thank you for this suggestion. We have added a sentence explaining that the databases were selected to provide complementary disciplinary coverage relevant to MLTC and ethnic inequalities, encompassing biomedical, social science, psychological, nursing and allied health research. Reviewer: Table 1. I’m not sure country of birth alone is necessarily an accurate indicator of ethnicity. Consider removing this or justify including this.

**Response:** Thank you for raising this important point. We agree that country of birth alone is not necessarily an accurate indicator of ethnicity. However, ethnicity is a multidimensional construct encompassing shared ancestry, cultural heritage, geographical origins and group identity. Accordingly, we consider Aboriginal and Indigenous identity to be relevant to the broader conceptualisation of ethnicity and have retained these classifications within our eligibility criteria. We have also retained studies reporting country of birth and migrant status to capture relevant evidence from countries where ethnicity data are not routinely collected or reported directly, and where migration related characteristics may be used to describe population groups. Importantly, we recognise that these measures are not equivalent to ethnicity. We have therefore clarified that country of birth and migrant status will be recorded separately as indicators of migration background, with findings from these studies synthesised separately from those reporting ethnicity or ethnic group identity.

# Reviewer report: Reviewer response for version 2

**DOI:** 10.21956/wellcomeopenres.30289.r165745

**Published:** 2026-09-22

**Authors:** Fabiola Eto

**Affiliations:** 1 Queen Mary University of London, London, England, UK

The protocol is clear, concise, and well-designed. I am already looking forward to reading the results.

 If possible at this stage, the authors should explain how they will harmonize different approaches to measuring SEP, especially across countries and healthcare settings. Alternatively, they should explain how they will account for these differences. 

 It will be interesting to see what the patterns look like in countries where access to health care varies more by insurance or ability to pay, especially for mental health conditions, in contrast with countries with a free, universal health system. These health care system differences should be considered when interpreting the findings, and how these would impact people from minority ethnic groups.

Is the study design appropriate for the research question?

Yes

Is the rationale for, and objectives of, the study clearly described?

Yes

Are sufficient details of the methods provided to allow replication by others?

Yes

Are the datasets clearly presented in a useable and accessible format?

Not applicable

Reviewer Expertise:

epidemiology

I confirm that I have read this submission and believe that I have an appropriate level of expertise to confirm that it is of an acceptable scientific standard.

# Response

**Published:** 2026-11-02

**Authors:** Irene Bossman

**Affiliations:** School of Health and Wellbeing, University of Glasgow College of Medical Veterinary and Life Sciences, Glasgow, Scotland, UK

**Response:** Thank you for this helpful comment and suggestion. We agree that differences in socioeconomic position (SEP) measures and healthcare systems are important when interpreting findings across countries and settings. Our SEP-related objective is to examine whether associations between ethnicity and MLTC prevalence or incidence vary by SEP, rather than to harmonise SEP measures internationally. We will therefore record the SEP indicators and categories used in each study, taking account of differences in their definitions and measurement across countries and settings. These differences will be considered when interpreting the findings and assessing patterns in the associations between ethnicity and MLTC prevalence or incidence across SEP groups. Findings will be described separately where differences in measurement or context limit meaningful comparisons. Subgroup analyses will be undertaken where sufficient studies with comparable measures are available.

# Reviewer report: Reviewer response for version 2

**DOI:** 10.21956/wellcomeopenres.30289.r166850

**Published:** 2026-09-17

**Authors:** Rachel Cooper

**Affiliations:** 1 Newcastle University, Newcastle upon Tyne, England, UK

Thank you for providing me with the opportunity to review this protocol which outlines plans to conduct a systematic review, following established guidelines, that identifies and synthesises published evidence on ethnic differences in multiple long-term conditions (MLTC).  This review will support efforts to better understand drivers of the stark inequalities in MLTC that are observed in the UK and other countries as required to aid identification of effective strategies to tackle these.

 It is noted that authors have already satisfactorily addressed comments provided by another reviewer.  My additional comments are as follows:

 1) The opening statement of the abstract “Multiple long-term conditions (MLTC) or multimorbidity is increasing among populations worldwide in the last decades.” Should be rephrased to improve flow and would benefit from the inclusion of a top-line definition of MLTC (assuming that some readers may not be aware that this relates to living with two or more long-term conditions).

 2) The authors state “This systematic review protocol plans to review the current literature to understand the incidence, patterns, prevalence, and impact of MLTC in different ethnic populations” – is it not actually the case that the authors will be examining differences between ethnic groups within populations?

 3) To ensure consistency of terminology I would recommend using the phrase ‘health condition’ rather than ‘illness’ throughout.

 4) The authors state ‘The effects of MLTC can vary among different minority ethnic groups. For example, people from certain minority ethnic groups (like South Asian) are more likely to develop long-term conditions like Type 2 diabetes compared to the white majority ethnic group in the UK.’  The example given here does not support the opening statement unless the implication is that there are varying effects of MLTC because of variation in the combinations of conditions most often experienced.

 5) Is ‘minority ethnic people’ an accepted term? – Should this be ‘people from minority ethnic groups’?

 6) It is not entirely clear how ethnicity will be considered in these questions **2. **What factors might be related to these MLTC? **3. **How often do people living with MLTC need hospital care?

 7) Please provide the citation that supports the statement that “One in three adults globally is living with two or more long-term conditions” and make clear that this is an estimate.

 8) As the argument is that we do not have sufficient evidence on ethnic differences in MLTC, I would suggest toning down the statement “MLTC is increasingly prevalent with age but has an early onset among people from minority ethnic backgrounds and socioeconomically deprived areas” by highlighting that there is some evidence to suggest this.

 9) Please check the protocol for grammatical errors and consider edits to improve flow – see for example “However, describing a population’s health in terms of ethnicity provide insights to disease causes affecting a particular ethnic group and help in designing targeted interventions and policies for health service planning.”

 10) Related to point 2, please consider revising the statement ‘across ethnic populations across the globe’ given what I think the authors plan to do is look at ethnic variation in various aspects of MLTC within different populations across the globe.

 11) Should “How do the incidence and prevalence of MLTC vary across ethnic populations?” read “How do the incidence and prevalence of MLTC vary by ethnicity within populations?”

 12) How many PPIE advisors were involved in the review?  What changes were made in response to their feedback?

 13) If the aim is to examine ethnic variation in incidence and prevalence of MLTC within different populations is it necessary to have ‘A study where majority of the study population are adults (aged 18 and above) living with multiple long-term conditions (MLTC).’ as an inclusion criterion?  Surely the observed variation in MLTC between ethnic groups would still be meaningful information even if the majority of the population are not living with MLTC.

 14) Given increasing adoption of the term MLTC in the UK, have the authors considered including this specific term within their searches?

Is the study design appropriate for the research question?

Partly

Is the rationale for, and objectives of, the study clearly described?

Partly

Are sufficient details of the methods provided to allow replication by others?

Partly

Are the datasets clearly presented in a useable and accessible format?

Not applicable

Reviewer Expertise:

Inequalities in multiple long-term conditions; life course epidemiology

I confirm that I have read this submission and believe that I have an appropriate level of expertise to confirm that it is of an acceptable scientific standard, however I have significant reservations, as outlined above.

# Response

**Published:** 2026-11-02

**Authors:** Irene Bossman

**Affiliations:** School of Health and Wellbeing, University of Glasgow College of Medical Veterinary and Life Sciences, Glasgow, Scotland, UK

We thank the reviewer for their constructive comments and suggestions on our systematic review protocol. We have carefully considered each point and revised the manuscript accordingly. Our responses to the individual comments are provided below, together with details of the corresponding amendments made to the manuscript.  

 1) The opening statement of the abstract “Multiple long-term conditions (MLTC) or multimorbidity is increasing among populations worldwide in the last decades.” Should be rephrased to improve flow and would benefit from the inclusion of a top-line definition of MLTC (assuming that some readers may not be aware that this relates to living with two or more long-term conditions).

 2) The authors state “This systematic review protocol plans to review the current literature to understand the incidence, patterns, prevalence, and impact of MLTC in different ethnic populations” – is it not actually the case that the authors will be examining differences between ethnic groups within populations?  

**Response to questions 1 and 2**: Thank you for these helpful comments. We have revised the opening statements of the abstract to improve flow and provide a concise definition of MLTC as the presence of two or more long term conditions in the same individual. We have also streamlined the subsequent sentences to clearly state the evidence gap and review aim. ‘Multiple long-term conditions (MLTC), also known as multimorbidity and commonly defined as two or more long term conditions in the same individual, are increasingly prevalent worldwide. Less is known about MLTC prevalence, incidence, patterns and health outcomes across ethnic groups. This systematic review protocol aims to examine ethnic differences in MLTC prevalence, incidence, patterns and health outcomes’.

 3) To ensure consistency of terminology I would recommend using the phrase ‘health condition’ rather than ‘illness’ throughout.

**Response: ** We have replaced ‘illness’ with ‘health condition’ in the plain language summary to ensure consistency as suggested.

 4) The authors state ‘The effects of MLTC can vary among different minority ethnic groups. For example, people from certain minority ethnic groups (like South Asian) are more likely to develop long-term conditions like Type 2 diabetes compared to the white majority ethnic group in the UK.’  The example given here does not support the opening statement unless the implication is that there are varying effects of MLTC because of variation in the combinations of conditions most often experienced.

**Response:** We agree that the original example did not directly support the statement that the effects of MLTC vary between ethnic groups. We have therefore revised the text to distinguish differences in the prevalence of individual long-term conditions from differences in MLTC. The revised text now states:  ‘We know that some long-term health conditions are more common in some ethnic groups than others. For example, research on individual long-term conditions has found that type 2 diabetes is more common among people from some ethnic groups, including people from South Asian backgrounds, than among people from the White ethnic group in the UK. But we know much less about how many people from different ethnic groups have two or more long-term health conditions at the same time. We also do not know enough about which conditions commonly occur together or whether people from different ethnic groups have different health outcomes…’

 5) Is ‘minority ethnic people’ an accepted term? – Should this be ‘people from minority ethnic groups’?

**Response:**

 Thank you for this suggestion. We have revised the wording throughout the protocol to use “people from minority ethnic groups” or “minority ethnic groups” rather than “minority ethnic people”. We agree that this is clearer and more appropriate terminology.

 6) It is not entirely clear how ethnicity will be considered in these questions **2. **What factors might be related to these MLTC? **3. **How often do people living with MLTC need hospital care?

**Response: ** We have revised the plain language summary to make it clearer how ethnicity will be considered in relation to each research question. In particular, the research questions now explicitly reflect our focus on understanding MLTC across different ethnic groups. For example, the highlighted questions have been revised as: “2. Does the link between ethnicity and MLTC vary according to age, sex or people's social and economic circumstances? 4. Do people from different ethnic groups with MLTC have different risks of needing healthcare or dying?”

 7) Please provide the citation that supports the statement that “One in three adults globally is living with two or more long-term conditions” and make clear that this is an estimate.

**Response**: Thank you for this helpful feedback. The text has been revised as below; A recent global systematic review and meta-analysis estimated that more than one in three adults living in the community have two or more long-term conditions (pooled prevalence: 37.2%), although estimates vary across populations and study methods (Chowdhury et al., 2023).

 8) As the argument is that we do not have sufficient evidence on ethnic differences in MLTC, I would suggest toning down the statement “MLTC is increasingly prevalent with age but has an early onset among people from minority ethnic backgrounds and socioeconomically deprived areas” by highlighting that there is some evidence to suggest this.

**Response: **Thank you for this helpful suggestion. We have revised the relevant sections of the introduction to better reflect the strength and limitations of the available evidence. The revised text now states: “Some evidence suggests that MLTC is increasingly prevalent with age, while its onset may occur earlier among people living in socioeconomically deprived areas and some minority ethnic groups.”

 9) Please check the protocol for grammatical errors and consider edits to improve flow – see for example “However, describing a population’s health in terms of ethnicity provide insights to disease causes affecting a particular ethnic group and help in designing targeted interventions and policies for health service planning.”

**Response:** Thank you for this helpful comment. We have carefully reviewed the entire protocol for grammatical errors, including the example highlighted, and have made the necessary revisions to improve clarity, consistency and overall flow throughout the protocol. For example, the highlighted text now reads “Examining population health by ethnicity can provide insights into the factors contributing to disease patterns within specific ethnic groups and can help inform targeted interventions and health service planning’. 10) Related to point 2, please consider revising the statement ‘across ethnic populations across the globe’ given what I think the authors plan to do is look at ethnic variation in various aspects of MLTC within different populations across the globe.

 11) Should “How do the incidence and prevalence of MLTC vary across ethnic populations?” read “How do the incidence and prevalence of MLTC vary by ethnicity within populations?”

**Response to questions 10-11: **Thank you for pointing out these errors. We have revised the highlighted phrases and sentences throughout the protocol to improve clarity and remove any ambiguity. For example, in response to comment 11, we have reworded the research question to read: “How do the incidence and prevalence of MLTC vary by ethnicity within populations?”

 12) How many PPIE advisors were involved in the review?  What changes were made in response to their feedback?

**Response**: Thank you for this question. We have clarified the number and roles of the PPIE advisors and members involved and have included the specific feedback provided by PPIE members and the changes made in response in the revised manuscript. The revised manuscript now begins with the following statement:

 “Our university based PPIE advisors with research expertise, supported community engagement and helped identify community and organisation leaders to support recruitment. A total of 10 PPIE members from diverse ethnic and migrant groups with lived experience of MLTC or as caregivers for people with MLTC were recruited from community groups ...”

 13) If the aim is to examine ethnic variation in incidence and prevalence of MLTC within different populations is it necessary to have ‘A study where majority of the study population are adults (aged 18 and above) living with multiple long-term conditions (MLTC).’ as an inclusion criterion?  Surely the observed variation in MLTC between ethnic groups would still be meaningful information even if the majority o-f the population are not living with MLTC.

**Response:** Thank you for raising this point. We agree that, for studies examining the incidence or prevalence of MLTC, participants may include adults both with and without MLTC, as this is necessary to estimate and compare incidence or prevalence across ethnic groups. This was clarified in the main protocol, which specified that studies examining the incidence or prevalence of MLTC could include adults with and without MLTC, whereas prognostic studies would include adults living with MLTC. We agree that this distinction was not made clear in the eligibility criteria table. We have now revised the table to clarify this distinction and ensure consistency with the protocol.

 14) Given increasing adoption of the term MLTC in the UK, have the authors considered including this specific term within their searches?

**Response:** Thank you for this helpful comment. Although the exact phrase “multiple long-term conditions” was not included as a standalone search term, our search strategy included a proximity search combining “multiple” with terms such as “condition* and “long-term or long term.” This allows “multiple” to be searched within two words of terms including “long-term”, “long term” and “condition*”, and therefore captures studies using the term “multiple long-term conditions”. We therefore retained the existing search strategy.

# Reviewer report: Reviewer response for version 2

**DOI:** 10.21956/wellcomeopenres.30289.r164699

**Published:** 2026-08-25

**Authors:** Atul Anand

**Affiliations:** 1 University of Edinburgh/BHF Centre for Cardiovascular Science, Edinburgh, UK

The authors have addressed my minor suggestions robustly. I have no further comments.

Is the study design appropriate for the research question?

Yes

Is the rationale for, and objectives of, the study clearly described?

Yes

Are sufficient details of the methods provided to allow replication by others?

Yes

Are the datasets clearly presented in a useable and accessible format?

Not applicable

Reviewer Expertise:

MLTC, ageing and frailty particularly using routine health data.

I confirm that I have read this submission and believe that I have an appropriate level of expertise to confirm that it is of an acceptable scientific standard.

# Reviewer report: Reviewer response for version 1

**DOI:** 10.21956/wellcomeopenres.27803.r155148

**Published:** 2026-05-27

**Authors:** Atul Anand

**Affiliations:** 1 University of Edinburgh/BHF Centre for Cardiovascular Science, Edinburgh, UK

This is a well described protocol in an important area of MLTC research. The authors are planning a systematic review +/- meta-analysis of ethnic differences in patterns and risk associated with MLTC, the incidence and prevalence of MLTC, and associations with age, sex and SEP.

 I have only minor comments and suggestions:

 1) There is some inconsistency in the use of the MLTC acronym - it is expanded twice in the abstract for example, and re-introduced at multiple places in the main text. This could be made more consistent.

 2) Complex multimorbidity is mentioned in the abstract but would benefit from a definition given the variation in the use of this term and need for the abstract to work independently of the main text.

 3) The version that I have been provided with for review had a truncated or incomplete plain language summary with only one research question described.

 4) The methods paragraph on 'Population' has an errant 'LTCs' at the end that needs removing.

 5) Given the aim of understanding global disease burden which is highly relevant for ethnicity, the decision to limit to English language publication requires a little more defence. It is maybe a pragmatic choice but this could be explained a little more.

 6) The authors have chosen to use the Newcastle-Ottawa Scale to assess risk of bias. The latest Cochrane guidance would suggest ROBINS-E. Again, this is not necessarily problematic, but this choice could be defended.

 7) Clearly meta-analysis potential cannot be determined at this stage, but it would have been good to see some criteria for where this would (or would not) be undertaken (e.g. minimum number of comparable studies).

Is the study design appropriate for the research question?

Yes

Is the rationale for, and objectives of, the study clearly described?

Yes

Are sufficient details of the methods provided to allow replication by others?

Yes

Are the datasets clearly presented in a useable and accessible format?

Not applicable

Reviewer Expertise:

MLTC, ageing and frailty particularly using routine health data.

I confirm that I have read this submission and believe that I have an appropriate level of expertise to confirm that it is of an acceptable scientific standard, however I have significant reservations, as outlined above.

# Response

**Published:** 2026-08-11

**Authors:** Irene Bossman

**Affiliations:** School of Health and Wellbeing, University of Glasgow College of Medical Veterinary and Life Sciences, Glasgow, Scotland, UK

We thank the Editor and the Reviewer for their constructive comments and suggestions of our systematic review protocol manuscript. We have revised the manuscript accordingly to incorporate the minor comments and suggestions offered, and have provided a response to each comment below:

 1) There is some inconsistency in the use of the MLTC acronym - it is expanded twice in the abstract for example and re-introduced at multiple places in the main text. This could be made more consistent.

**Response:** The new version uploaded has addressed the repetitive expansion of the MLTC acronym in the abstract and full text accordingly.

 2) Complex multimorbidity is mentioned in the abstract but would benefit from a definition given the variation in the use of this term and need for the abstract to work independently of the main text.

**Response:** We have provided a definition of complex multimorbidity in the abstract as suggested.

 3) The version that I have been provided with for review had a truncated or incomplete plain language summary with only one research question described.

**Response:** Thank you for highlighting this issue. The plain language summary submitted with our manuscript was complete, so this appears to have occurred during the editorial or publishing process rather than in the submitted version. We will bring this to the attention of the editorial team to ensure that the complete plain language summary is included.

 4) The methods paragraph on 'Population' has an errant 'LTCs' at the end that needs removing.

**Response:** We have removed this errant ‘LTCs’ from the end of that paragraph.

 5) Given the aim of understanding global disease burden, which is highly relevant for ethnicity, the decision to limit to English language publication requires a little more defense. It is maybe a pragmatic choice, but this could be explained a little more.

**Response:** Thank you for this helpful suggestion. We have clarified in the new version of the protocol to state that our literature search will not be restricted by language. Where potentially eligible studies for screening are not in English, they will be translated as appropriate to determine eligibility.

 6) The authors have chosen to use the Newcastle-Ottawa Scale to assess risk of bias. The latest Cochrane guidance would suggest ROBINS-E. Again, this is not necessarily problematic, but this choice could be defended.

**Response: **Thank you for highlighting the need to further justify the use of the Newcastle–Ottawa Scale (NOS). We have clarified that the adapted NOS was selected because this review focuses on the prevalence, incidence, and health outcomes of multimorbidity across ethnic groups. These objectives are descriptive and observational, aiming to examine patterns and associations rather than causal effects. As ROBINS-E is designed for assessing risk of bias in studies intended to support causal inference, we considered the adapted NOS to be the more appropriate tool for assessing the quality of the included studies in our review.

 7) Clearly meta-analysis potential cannot be determined at this stage, but it would have been good to see some criteria for where this would (or would not) be undertaken (e.g. minimum number of comparable studies).

**Response: **We have further clarified the criteria for undertaking a meta-analysis in the revised protocol version. Specifically, we state that a meta-analysis will be conducted where at least two studies are sufficiently comparable in terms of the exposure, study design, and outcome measures.

 We thank Dr. Anand for taking the time to review our manuscript and for their constructive feedback.
